# Editorial: Symmetry as a guiding principle in artificial and brain neural networks, volume II

**DOI:** 10.3389/fncom.2024.1527725

**Published:** 2024-12-09

**Authors:** Fabio Anselmi, Ankit B. Patel

**Affiliations:** ^1^Department of Mathematics, Informatics and Geoscience, University of Trieste, Trieste, Italy; ^2^Department of Electrical and Computer Engineering, Rice University, Houston, TX, United States; ^3^Center for Neuroscience and Artificial Intelligence, Baylor College of Medicine, Houston, TX, United States

**Keywords:** symmetry, machine learning (ML), computational neuroscience, implicit bias, neural networks

As a follow-up to our previous editorial, this Research Topic further delves into the the understanding of how symmetries shape information processing in both biological and artificial neural networks. While the prior Research Topic focused on the foundational role of symmetries in sensory input and its organization in neural systems, this editorial, beside continuing that topic, presents new research on the mechanisms behind symmetry-driven representations and their robustness, particularly in artificial neural networks.

Indeed, symmetry plays a pivotal role in simplifying the complexity of input data and promoting robustness in neural networks. In both artificial systems and the brain, symmetry helps create efficient representations that generalize well to unseen data and reduce the burden of learning from large datasets. By leveraging invariant and equivariant properties of sensory data, neural networks–both biological and artificial–can enhance their ability to interpret and respond to the world around them. This Research Topic further explores the intersection of symmetries, learning dynamics, and neural representations in both artificial and biological systems.

The first contribution, DiTullio et al. delves into how the brain may use time as a supervisory signal to learn auditory features. By exploring the natural regularities and symmetries in the auditory domain, the authors propose that temporal consistency is key to learning auditory object representations, particularly in cluttered environments. The work demonstrates that models capturing these temporal regularities outperform conventional feature-selection algorithms such as principal component analysis (PCA) and independent component analysis (ICA) in auditory discrimination tasks. The implications for both neuroscience and machine learning are profound, suggesting that the temporal structure of stimuli provides an essential basis for efficient sensory processing and generalization.

Vision is another sensory modality where symmetry plays a pivotal role. The article (Lindeberg) presents a theoretical framework for understanding the geometric properties of visual receptive fields in the brain. Covariance, or equivariance, ensures that the transformation of sensory input results in a corresponding transformation in the neural representation. The study of these properties reveals how visual receptive fields in the primary visual cortex (V1) are tuned to transformations such as spatial scaling and Altemporal scaling. The authors argue that these symmetry properties provide the biological vision system with robustness to the variations encountered in natural environments, opening new avenues for biologically inspired machine vision systems that mimic these neural mechanisms.

Symmetry also governs higher-level perceptual tasks, such as three-dimensional (3D) shape reconstruction from visual input. Beers and Pizlo explores how the human visual system uses symmetry, specifically mirror symmetry and 3D compactness, to reconstruct 3D shapes from two-dimensional images. The study's computational models, validated by human performance in psychophysical tasks, suggest that secondary mirror symmetry plays a crucial role in enabling accurate 3D perception. This work highlights the importance of symmetry as a computational principle in both biological and artificial vision systems and contributes to our understanding of the neural mechanisms underlying 3D object recognition.

The last two papers delve into how symmetries relate to an artificial neural network representation and its robustness. In specific, the article (Ferrari et al.) introduces a novel class of operators, called P-GENEOs, that enable the encoding of partial equivariance in neural networks. This approach allows networks to respect specific symmetry transformations without requiring full equivariance. By formalizing these operators using topological spaces and pseudo-metrics, the authors provide a new perspective on how neural networks can achieve flexible and efficient learning of symmetries. This theoretical advance has potential applications in a variety of domains, from computer vision to the analysis of complex biological data.

The final contribution in this Research Topic, Caro et al., explores the relationship between symmetry in convolutional operations and adversarial vulnerability in neural networks. The authors propose that the translational symmetry in convolutional layers biases networks toward learning high-frequency features, making them more susceptible to adversarial attacks. By analyzing the impact of convolutional kernel sizes and architectural choices on adversarial robustness, the study demonstrates that changing this symmetry by using larger kernels or breaking it by adopting non-convolutional architectures such as vision transformers, reduces this vulnerability. The work underscores the critical role of implicit architectural biases in the generalization and robustness of neural networks.

All together the contributions in this Research Topic highlight that symmetry not only facilitates efficient information processing by reducing the complexity of sensory input (in natural and artificial networks) but also plays a crucial role in robustness to adversarial perturbations. Thus, by understanding how neural networks encode, learn, and exploit symmetries, we can gain deeper insights into the principles governing intelligence. We hope that this Research Topic will inspire further research into the interplay between symmetry, learning, and neural representations, ultimately advancing our understanding of both natural and artificial intelligence.

